# Decreased NK Cell FcRγ in HIV-1 Infected Individuals Receiving Combination Antiretroviral Therapy: a Cross Sectional Study

**DOI:** 10.1371/journal.pone.0009643

**Published:** 2010-03-10

**Authors:** Edwin Leeansyah, Jingling Zhou, Geza Paukovics, Sharon R. Lewin, Suzanne M. Crowe, Anthony Jaworowski

**Affiliations:** 1 Centre for Virology, The Macfarlane Burnet Institute for Medical Research and Public Health, Melbourne, Australia; 2 Department of Medicine, Monash University, Melbourne, Australia; 3 Infectious Diseases Unit, The Alfred Hospital, Melbourne, Australia; Karolinska Institutet, Sweden

## Abstract

**Background:**

FcRγ is an immunoreceptor tyrosine-based activation motif (ITAM)-signalling protein essential for immunoreceptor signaling and monocyte, macrophage and NK cell function. Previous study from our laboratory showed that FcRγ is down-regulated in HIV-infected macrophages in vitro. FcRγ expression in immune cells present in HIV-infected individuals is unknown.

**Methodology/Principal Findings:**

We compared FcRγ expression in peripheral blood mononuclear cells isolated from HIV-1-infected individuals receiving combination antiretroviral therapy and healthy, HIV-1-uninfected individuals. FcRγ mRNA and protein levels were measured using quantitative real-time PCR and immunoblotting, respectively. CD56^+^ CD94^+^ lymphocytes isolated from blood of HIV-1 infected individuals had reduced FcRγ protein expression compared to HIV-uninfected individuals (decrease = 76.8%, n = 18 and n = 12 respectively, p = 0.0036). In a second group of patients, highly purified NK cells had reduced FcRγ protein expression compared to uninfected controls (decrease = 50.2%, n = 9 and n = 8 respectively, p = 0.021). Decreased FcRγ expression in CD56+CD94+ lymphocytes was associated with reduced mRNA (51.7%, p = 0.021) but this was not observed for the smaller group of patients analysed for NK cell expression (p = 0.36).

**Conclusion/Significance:**

These data suggest biochemical defects in ITAM-dependent signalling within NK cells in HIV-infected individuals which is present in the context of treatment with combination antiretroviral therapy.

## Introduction

HIV-1 infection, like other chronic inflammatory diseases, is associated with immune activation, which is an important independent predictor of HIV-1 disease progression [1], [2]. Immune activation may be caused by a combination of persistent HIV-1 replication and innate immune activation via gut-associated pathogens, associated with loss of gut-associated lymphoid tissue (GALT) [3] Immune activation markers on leukocytes and in plasma may persist even after initiation of combination antiretroviral therapy (cART) [4], [5], [6]. The observation that GALT is not as rapidly restored as peripheral blood CD4 T cell counts following cART [7], [8] suggests that immune activation due to elevated bacterial products and its downstream consequences also persist after viral suppression, which is indicated by elevated endotoxin levels in plasma even after 48 weeks of cART [3].

Chronic inflammatory diseases are characterised by defective immunoreceptor signaling mediated by immunoreceptor tyrosine-based activation motif (ITAM) domain-containing adaptor proteins (reviewed in [9]). The only reports of ITAM protein expression in the context of HIV infection have been older observations that HIV-1 infection in patients not receiving cART was associated with decreased expression of TCRζ (an ITAM-signalling protein that mediates T-cell receptor signaling) in bystander cells that are not necessarily infected by HIV-1, including NK and CD8^+^ T cells [10], [11], [12]. Limited data suggest that expression of TCRζ is restored by cART [10].

Fc receptor common γ-chain (FcRγ) is the most widespread ITAM-signaling protein. It is broadly expressed in haematopoietic cells including monocytes, macrophages, NK cells and effector CD4^+^ T cells. It is promiscuously associated with, and transduces signals from, a broad range of immunoreceptors, including FcαR, FcεR, FcγR, NKp46 and TCR [13]. Studies using NK cells from FcRγ knockout mice show that this protein is absolutely required for NK cell mediated cytotoxicity [14]. We have previously shown that FcRγ expression is decreased in human monocyte-derived macrophages (MDM) infected with HIV-1 *in vitro*, leading to impaired FcγR-mediated phagocytosis in this model of tissue macrophages [15], [16]. This inhibition is not restricted to HIV-1-infected cells [16] suggesting that HIV-1 infection reduces FcRγ expression by a bystander mechanism. Whether HIV reduces FcRγ in other cell types is not known. There have been no studies examining expression of FcRγ in chronic inflammatory diseases, and in the context of HIV-1 infection in particular. Given the importance of FcRγ in signaling from a wide variety of immunoreceptors, and our *in vitro* observations that HIV-1 infection causes a bystander-mediated decrease in FcRγ expression, we examined its expression in peripheral blood mononuclear cells from a cohort of HIV-1 infected patients receiving cART.

## Methods

### Ethics Statement

This study is approved by The Alfred Human Research Ethics Committee (Project 36/02) with a protocol that conforms to the provisions of the Declaration of Helsinki (as revised in Edinburgh 2000).

### Objectives

To measure FcRγ expression in peripheral blood cells from a current cohort of HIV-infected and control, HIV-uninfected subjects. We hypothesise that HIV-infection is associated with decreased expression of FcRγ in the context of treatment with combination antiretroviral therapy (cART).

### Participants

All HIV-infected individuals were recruited from the Alfred Hospital Infectious Diseases Outpatient Clinic (Melbourne, Australia) and were receiving cART with no history of, or current, AIDS-defining illness at recruitment. Blood samples were collected by venepuncture into EDTA containing blood collection tubes with informed written consent. Gender-matched, healthy HIV-uninfected individuals were recruited via advertisement within the Burnet Institute (Melbourne, Australia) and blood was collected with ethics approval and written informed consent by venepuncture into EDTA tubes.

### Antibodies

Mouse anti-GAPDH mAb (clone 6C5) was from Santa Cruz Biotechnology, and rabbit anti-FcRγ antiserum was from Dr. Bruce Wines (Burnet Institute, Melbourne, Australia). Mouse fluorochrome-conjugated antibodies CD3-FITC, CD14-PE, CD14-FITC, CD16-PerCP-Cy5.5, CD19-FITC, CD56-APC, CD94-APC and isotype matched IgG_1_-FITC, -PE, -PerCP-Cy5.5 and -APC were from BD Biosciences. Goat anti-rabbit Alexa Fluor® 680 and goat anti-mouse IRDye™ 800 were from Molecular Probes and Rockland Immunochemicals, respectively.

### Cell Isolation

Peripheral blood mononuclear cells (PBMC) were labeled with 25 µg/ml anti-CD3-FITC and anti-CD14-PE, or 37.5 µg/ml of anti-CD56-APC and anti-CD94-APC or with corresponding mouse IgG_1_ isotype control antibodies. Cells were washed twice in cold FACS sorting buffer (Ca^2+^,Mg^2+^ free PBS, 2 mM EDTA, 1% newborn calf serum (HyClone)) and resuspended at 4×10^7^ cells/ml. CD14^+^ monocytes, CD3^−^ CD56^+^/CD94^+^ NK cells, and CD3^+^ T lymphocytes were isolated using a high-speed cell sorter (FACSAria™, BD Biosciences) under PC3 containment conditions. In selected experiments, PBMC were stained with 25 µg/ml each of anti-CD3-FITC, anti-CD14-FITC and anti-CD19-FITC, 37.5 µg/ml of each anti-CD16-PerCP-Cy5.5 and anti-CD56-APC in combination. NK cells (gated on CD3^−^/CD14^−^/CD19^−^ cells in the lymphocyte region) were subsequently sorted into CD56^dim^ CD16^+^ and CD56^bright^ CD16^−^ subsets. Sorted cells were washed twice in cold Ca^2+^,Mg^2+^-free PBS, and 10^5^ cells from each population were lysed with RNA lysis/binding buffer [16]. Remaining cells were lysed with 200 µl cold Triton lysis buffer (TLB) [16]. TLB extracts were incubated at 4°C for 10 min and clarified at 20,000×*g* for 10 min at 4°C.

### Measurement of FcRγ and TCRζ mRNA Expression

FcRγ mRNA was measured by quantitative real-time PCR (Q-PCR) as described [16]. TCRζ (GenBank accession NM198053) mRNA was measured under identical conditions but using the forward primer 5′-TCAGCCTCTGCCTCCCAGCCTCTTTCT-3′ and reverse primer 5′-CTCACTGTAGGCCTCCGCCA-3′. Both FcRγ and TCRζ mRNA levels were quantified using the comparative threshold method, with GAPDH mRNA as internal standard. FcRγ was co-immunoprecipitated from cellular extracts with GAPDH as an internal control then immunoprecipitates were resolved by SDS PAGE. Blots were probed with rabbit anti-FcRγ plus mouse anti-GAPDH then goat anti-rabbit Alexa Fluor® 680 plus goat anti-mouse IRDye™ 800. Fluorescence in both channels was quantified using a LI-COR Odyssey infrared (IR) imager (LI-COR Biosciences) and expressed as a ratio of FcRγ∶GAPDH fluorescence.

### Statistical Analysis

Statistical significance between uninfected and HIV-1-infected groups was calculated using Mann-Whitney non-parametric U test. Spearman's rank test for non-parametric data was used to determine correlations. All statistical analyses were carried out using Prism 5.0 software (GraphPad Software). Significance was assumed when probability values were <0.05.

## Results

**Figure 1:**
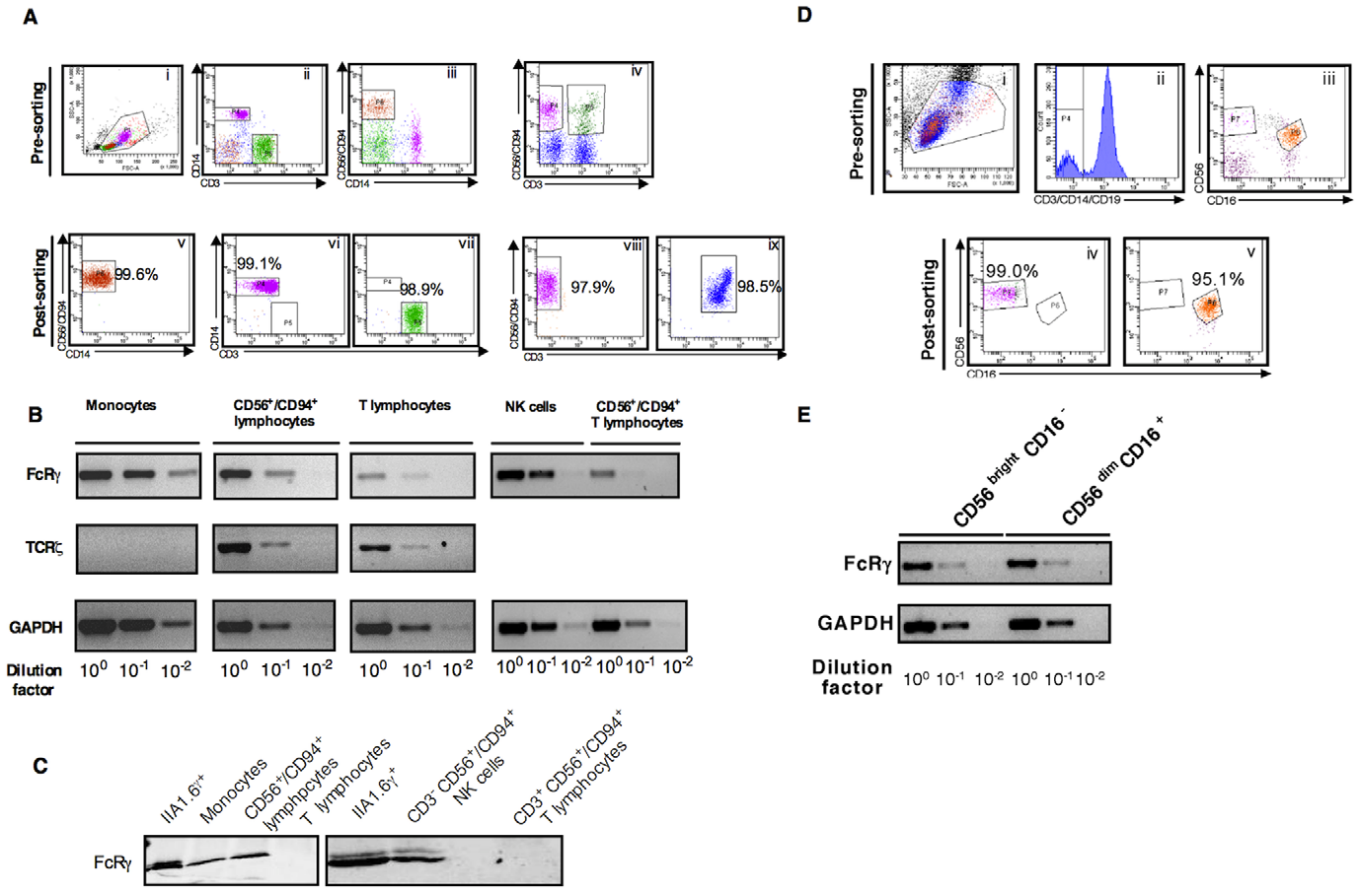
FcRγ and TCRζ expression in human peripheral blood mononuclear cell populations. (A). Monocytes (CD3^−^ CD14^+^ cells, ii), CD3^−/+^ CD56^+^/CD94^+^ cells (a mixture of CD56^+^/CD94^+^ T lymphocytes and NK cells, iii), T lymphocytes (CD3^+^ cells, ii) and pure NK cells and CD56^+^/CD94^+^ T lymphocytes (CD3^−^ CD56^+^/CD94^+^ cells, and CD3^+^ CD56^+^/CD94^+^ cells respectively, iv) were isolated from PBMC (i) of an HIV-negative donor, by FACS to >97% purity for each population (v-ix). (B) FcRγ, TCRζ and glyceraldehyde-3-phosphate dehydrogenase (GAPDH) mRNA levels were measured by conventional PCR using serial 10-fold dilutions of cell extract as indicated. (C). FcRγ expression was measured by immunoblotting in protein extracts from the indicated cell populations purified by FACS. (D). Lymphocytes were initially gated from PBMC of a healthy, HIV-uninfected donor using light scatter properties (i) then NK cells gated as CD3^−^ CD14^−^ CD19^−^ cells (ii). Gates were established for NK cell subsets (CD56^bright^CD16− and CD56^dim^CD16+, (iii)) which were then sorted by FACS to 99.0% and 95.1% purity respectively (iv and v). (E). FcRγ and GAPDH mRNA expression in each NK cell subset were determined by conventional PCR using serial 10-fold dilutions of cell extract as indicated.

RNA extracts prepared from a single HIV-uninfected donor were analysed by PCR. FcRγ mRNA was expressed at high levels in monocytes and moderate levels in NK cells, but low levels in T lymphocytes (Figure 1A–C). Within CD56/CD94-positive cells most of the expression was found within CD3^−^ NK cells and only minor expression within the CD3^+^ population. TCRζ was not detected in monocytes (Figure 1B,C). Protein analysis also showed a lack of FcRγ expression in T lymphocytes either within the whole CD3^+^ population or specifically within CD56^+^/CD94^+^ T lymphocytes (Figure 1C). FcRγ mRNA expression was similar in both the CD56^bright^CD16− and CD56^dim^CD16+NK subsets (Figure 1D,E). In the following experiments, FcRγ expression was therefore measured in monocyte and NK cell lysates and TCRζ expression in T cell and NK cell lysates.

**Figure 2:**
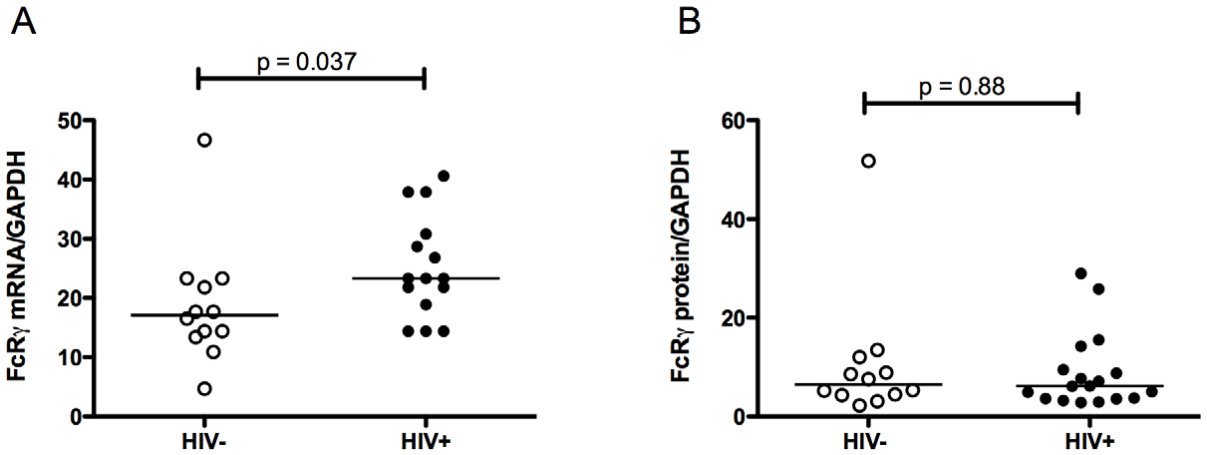
FcRγ expression in monocytes of HIV-infected and uninfected individuals. FcRγ mRNA (A) and protein (B) were determined by Q-PCR and quantitative infrared immunoblotting in extracts from monocytes purified by FACS from blood of HIV-1 infected (individuals 1–18, Table 1) and control subjects. Fluorescence from FcRγ immunoblots measured at 680 nm was normalised to fluorescence of GAPDH in the same immunoblots, measured at 800 nm. FcRγ mRNA was determined from real-time PCR measurements using the comparative threshold method with GAPDH mRNA serving as internal control. Differences between groups were tested using the Mann-Whitney U test for non-parametric data, with a value <0.05 assumed to be significant. Horizontal bars represent median values.

**Figure 3:**
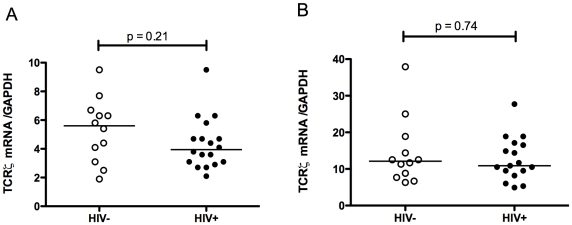
TCRζ expression in CD56+/CD94+ and CD3+ T lymphocyte populations of HIV-infected and uninfected individuals. TCRζ mRNA in T lymphocytes (A) and CD56^+^/CD94^+^ lymphocytes (B) purified by FACS from blood of HIV-1 infected (individuals 1–18, Table 1) and control subjects was measured by Q-PCR using the comparative threshold method as in Figure 2A. The Mann-Whitney U test was used to assess statistical significance. Horizontal bars represent median values.

Monocytes and T lymphocytes were purified from blood sampled from eighteen HIV-infected patients (subjects 1–18, Table 1) and twelve healthy, HIV-uninfected individuals. All patients received cART at time of recruitment and most (78%) had viral load (VL)<50 copies/ml. Monocytes from HIV-infected individuals showed a small but significant increase in FcRγ mRNA (median HIV+ = 23.30, HIV− = 17.10, p = 0.037, Figure 2A) compared to that from uninfected controls, although similar levels of FcRγ protein were expressed (median HIV+ = 6.155, HIV− = 6.465, p = 0.88, Figure 2B). TCRζ mRNA expression in T lymphocytes was unchanged in these patients (median HIV+ = 3.95, HIV− = 5.60, p = 0.21, Figure 3A) consistent with previous findings that TCRζ expression is restored following cART [10]. Analysis of CD56^+^/CD94^+^ cells from the sorted populations (consisting of NK cells plus CD56^+^/CD94^+^ lymphocytes) also showed similar levels of TCRζ expression (median HIV+ = 10.9, HIV− = 12.1, p = 0.74, Figure 3B).

**Table 1:** Clinical details of HIV-1 infected patients used in this study.

Donor	CD4 (cells/µl)	Nadir CD4 (cells/µl)	Viral load (RNA copies/ml)	Current therapy regimen^1^
1	640	40	8300	3TC, TDF, ATV, T20,
2	396	143	<50	FTC, TDF, FPV
3	57	54	6750	3TC, ABC, FPV
4	599	50	<50	3TC, AZT, ATV
5	178	140	<50	FTC, TDF, NVP,
6	381	0	1600	DRV, ETV, 3TC, RTV
7	243	72	>100,000	3TC, D4T, TDF, DRV, T20
8	830	110	<50	3TC, ABC, LPV
9	352	54	<50	3TC, ABC, ATV
10	430	0	<50	FTC, TDF, LPV
11	598	230	<50	FTC, TDF, RGV, T20
12	728	231	<50	3TC, ABC,NVP
13	365	258	<50	FTC, TDF, EFV
14	381	381	<50	3TC, ABC, NVP
15	891	315	<50	3TC, AZT, NVP
16	639	252	<50	FTC, TDF, NVP,
17	1205	308	<50	FTC, TDF, EFV
18	1176	230	<50	FTC, TDF, NVP
19	255	149	<50	3TC, TDF, NVP
20	275	67	1700	FTC, TDF, EFV
21	720	97	<50	3TC, TDF, NVP
22	15	15	>100,000	AZT, ABC, 3TC, ETV, RGV
23	179	139	900	TDF, ABC, 3TC, ETV, RGV
24	703	100	<50	FTC, TDF, NVP
25	250	89	350	TDF, FTC, NVP
26	802	376	<50	AZT, 3TC, NVP
27	172	40	<50	AZT, 3TC, NVP

1 3TC: lamivudine, ABC: abacavir, ATV: atazanavir, AZT: zidovudine, D4T: stavudine, DRV: darunavir, EFV: efavirenz, ETV: etravirine, FPV: fosamprenavir, FTC: emtricitabine, LPV: lopinavir, NVP: nevirapine, RGV: raltegravir, RTV: ritonavir, T20: enfuvirtide, TDF: tenofovir.

**Figure 4:**
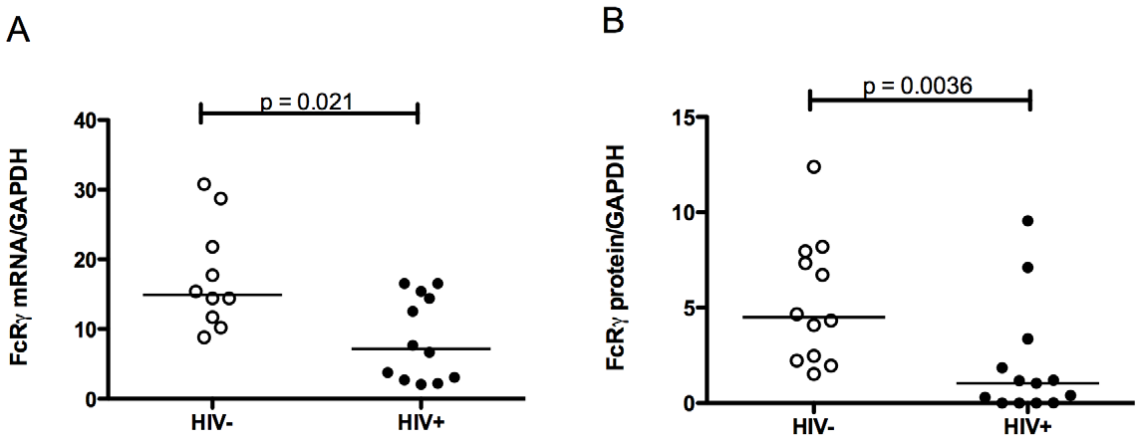
FcRγ expression in CD56^+^/CD94^+^ lymphocytes of HIV-infected and uninfected individuals. FcRγ mRNA (A) and protein (B) were measured in FACS-sorted CD56^+^/CD94^+^ lymphocytes from blood of HIV-1 infected (individuals 1–18, Table 1) and control subjects by Q-PCR using the comparative threshold and quantitative immunoblotting method as described. The Mann-Whitney U test was used to assess statistical significance. Horizontal bars represent median values.

**Figure 5:**
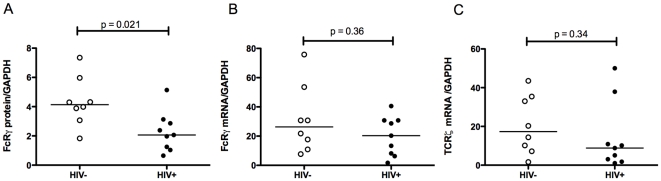
FcRγ and TCRζ expression in NK cells of HIV-infected and uninfected individuals. NK cells purified from blood of an additional 9 HIV-infected (individuals 19–27, Table 1) and 8 control subjects. FcRγ protein (A) and mRNA (B) and TCRζ mRNA (C) were measured in FACS-sorted CD3^−^ CD56^+^/CD94^+^ NK cells using quantitative immunoblotting and Q-PCR as described. The Mann-Whitney U test was used to assess statistical significance. Horizontal bars represent median values.

When FcRγ expression was compared, however, the CD56^+^/CD94^+^ cell population showed a considerable decrease in both FcRγ mRNA expression (median HIV+ = 7.2, HIV− = 14.9, p = 0.021, Figure 4A) and protein expression (median HIV+ = 1.040, HIV− = 4.490, p = 0.0036, Figure 4B). Since within this mixed population only the CD3^−^CD56^+^/CD94^+^ NK cell fraction expressed appreciable FcRγ (Figure 1B,C) this suggests that decreased FcRγ expression in HIV-infected individuals occurs in NK cells. To address this, a further nine HIV-infected patients (subjects 19–27, Table 1) and eight control subjects were enrolled and PBMC prepared from these individuals were specifically sorted to obtain highly purified monocytes and NK cells. There was a 50% decrease in FcRγ protein expression (median protein HIV+ = 2.061, HIV− = 4.139, p = 0.021, Figure 5A) but FcRγ mRNA expression was unchanged (median RNA HIV+ = 20.30, HIV− = 26.30, p = 0.36, Figure 5B) in NK cells from HIV-infected individuals. Levels of TCRζ mRNA in NK cells were equivalent in the two populations (median mRNA HIV+ = 8.80, HIV− = 17.35, p = 0.34, Figure 5C) again consistent with previous reports [10]. There was no significant correlation between decreased FcRγ protein levels in NK cells with either nadir or current CD4 counts (r = −0.22, p = 0.58 and r = 0.18, p = 0.64 respectively) from these HIV-1-infected individuals (n = 9, patients 19–27). Since only one patient (patient 22, Table 1) had high level of viremia, determining whether levels of FcRγ correlated with viral load was not possible. However since five of the nine patients examined had an undetectable viral load, it is not likely that viremia is a significant contributor to reduced NK cell FcRγ expression in the context of HIV infection.

**Figure 6:**
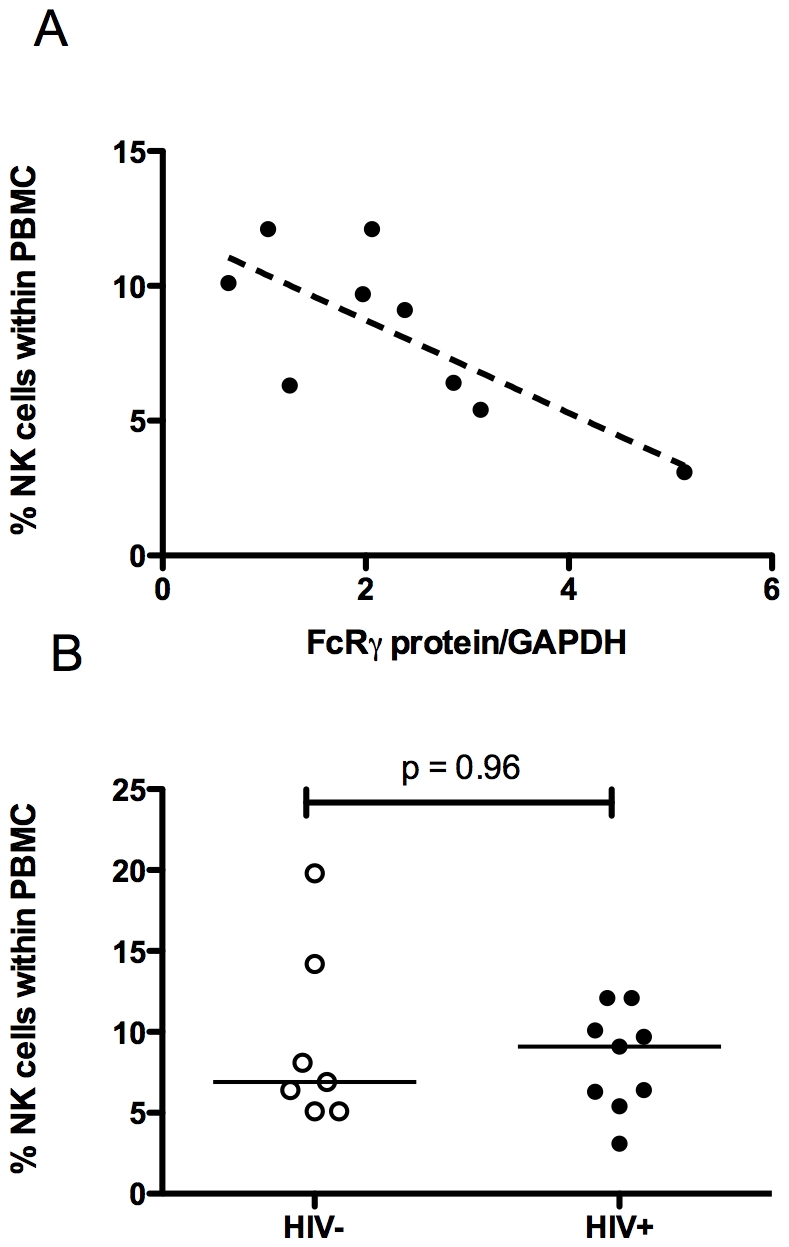
Correlation of NK cell proportions with FcRγ expression in HIV-infected individuals. (A) Correlation between NK cell FcRγ protein expression and the proportion of NK cells in patient PBMC (donors 19–27) measured by flow cytometry. (B) Proportion of NK cells in PBMC (individuals 19–27, Table 1) measured by flow cytometry. The Mann-Whitney U test was used to assess statistical significance. Horizontal bars represent median values.

Chronic HIV-1 infection is associated with a progressive decline in NK cell numbers and function [17]. We therefore examined the potential association between FcRγ expression and NK cell proportion in peripheral blood mononuclear cells. Unexpectedly, there was a strong negative correlation between FcRγ protein levels and the proportion of NK cells in HIV-infected individuals (Spearman's r = −0.71, p = 0.037, Figure 6A) although the proportion of NK cells was not significantly different in this cohort of cART treated patients compared to uninfected control subjects (Figure 6B) in agreement with results from others [18]. Similarly, we observed a negative correlation between FcRγ mRNA concentration and proportion of NK cells in these individuals (r^2^ = 0.52, p = 0.028, data not shown). There was no correlation between FcRγ expression and NK cell frequency in HIV-uninfected subjects at both mRNA and protein levels (r^2^ = 5×10^−5^, p = 0.99 and r^2^ = 0.024, p = 0.71, respectively). In this group of patients, therefore, decreased FcRγ expression is principally found in HIV-1-infected individuals with preserved NK cell numbers. It is not likely that decreased FcRγ expression is due to a shift in the proportion of CD56^dim^ CD16^+^ NK and CD56^bright^ CD16^−^ cell subsets since both subsets express similar levels of FcRγ (Figure 1E).

## Discussion

We have shown that FcRγ mRNA and protein expression in HIV-infected individuals receiving cART is decreased in CD56^+^/CD94^+^ lymphocytes in which the major FcRγ-expressing cells are NK cells, but are not decreased in monocytes. We confirmed that the decrease occurs in NK cells using cells purified from a second group of patients also receiving cART. Decreased FcRγ expression was due to loss of FcRγ expression at a single cell level, and not due to depletion of FcRγ-expressing NK cells, or altered proportions of NK cell subsets within the CD56^+^/CD94^+^ population of our cohort of HIV-infected individuals. FcRγ protein expression within monocytes is not altered, suggesting that the defect in ITAM protein expression is specific for FcRγ in NK cells. This is also suggested by the observation that TCRζ mRNA levels were not decreased in either T lymphocytes or NK cells purified from these individuals. This is the first report of expression of the important ITAM signalling protein FcRγ in peripheral blood mononuclear cells from patients with HIV-1 infection or, to our knowledge, any viral infection.

Since FcRγ expression in human immune cells is not well documented, it was initially measured in monocytes, T lymphocytes and CD56^+^/CD94^+^ lymphocytes obtained from uninfected individuals and showed high levels of expression of both mRNA and protein in monocytes and CD56^+^/CD94^+^ lymphocytes. Within the CD56^+^/CD94^+^ lymphocytes population, FcRγ was only detected in NK cells but not in CD56/CD94-expressing T lymphocytes (Figure 1). This observation suggests that FcRγ expression detected in CD56^+^/CD94^+^ lymphocytes was due to FcRγ-expressing NK cells. Given that FcRγ also acts as a chaperone and is required for CD16 surface expression [19], [20], [21], [22], the levels of FcRγ in CD56^dim^ CD16^+^ and CD56^bright^ CD16^−^ NK cell subsets were compared. FcRγ mRNA expression in CD56^dim^ CD16^+^ and CD56^bright^ CD16^−^ NK cell subsets were however similar, which suggests that FcRγ levels are not limiting CD16 surface expression in the latter population. To our knowledge, this is the first study to investigate FcRγ expression in NK cell subsets. Our data also show that there is little or no FcRγ expression in T lymphocytes compared to monocytes and CD56^+^/CD94^+^ lymphocytes in either HIV-1-infected or uninfected individuals (data not shown).

In a limited number of patients, we observed an increase in FcRγ mRNA expression in monocytes relative to HIV-uninfected subjects. This was not observed however with FcRγ protein levels, which were similar to those of uninfected individuals. The significance of increased FcRγ mRNA levels in monocytes from HIV-1-infected individuals is unclear and will need to be confirmed in a larger cohort of patients. In contrast to monocytes, we observed a significant decrease in FcRγ mRNA and protein expression in CD56^+^/CD94^+^ lymphocytes of HIV-infected individuals. Our data demonstrate that decreased FcRγ mRNA expression was strongly and significantly correlated with decreased FcRγ protein expression within the mixed CD56^+^/CD94^+^ lymphocyte population isolated from HIV-1-infected individuals. We did not observe any significant correlation of defective FcRγ mRNA and protein with nadir or current CD4 counts. This contrasts with the previous reports on the effect of HIV-1 infection on TCRζ expression in patients not receiving cART in which there was a correlation with CD4 counts and an inverse correlation with viral loads [10], [11], [23]. Since only NK cells within this population express FcRγ, we considered whether FcRγ depletion within CD56^+^/CD94^+^ lymphocytes may be caused by the loss of FcRγ-expressing NK cells. However, there was no correlation between decreased FcRγ mRNA or protein levels and the proportion of NK cells within the sorted CD56^+^/CD94^+^ populations analysed (data not shown). This shows that decreased FcRγ levels in CD56^+^/CD94^+^ population is more likely due to loss of FcRγ expression within NK cells. We confirmed this by measuring FcRγ expression in purified NK cells from additional patients and control subjects. FcRγ mRNA levels were not significantly decreased in NK cells from these additional patients. When we investigated the expression of FcRγ as a function of age within the HIV-uninfected donors there was no correlation at either the protein (r^2^ = 0.055, p = 0.34) or the mRNA level (r^2^ = 0.015, p = 0.63). The difference between in FcRγ expression between HIV-infected and uninfected subjects is therefore not due to differences in age. Analysis of NK cells from a larger numbers of patients is required to determine whether decreased FcRγ mRNA occurs in patients receiving cART. Our ongoing study is currently addressing this.

Several studies have shown that the proportion of NK cell subsets is altered during chronic HIV-1 infection, with loss of CD56^+^ CD16^−/+^ NK cells and accumulation of functionally anergic CD56^−^ CD16^+^ NK cell subsets [17], [24], [25], [26]. However, in HIV-1 infection where viral replication is well-controlled, the proportion of these subsets is similar to that of uninfected individuals [17], [24]. Since the majority of HIV-1-infected individuals in our cohort had undetectable viral loads and we show that both CD56^dim^ CD16^+^ and CD56^bright^ CD16^−^ NK cells expressed equal levels of FcRγ, alterations in the proportion of these subsets are unlikely to account for the FcRγ depletion observed in our study. Taking all of these observations together, our results indicate that decreased FcRγ levels are likely to be due to loss of FcRγ expression within FcRγ-expressing NK cells.

The potential mechanisms underlying defective FcRγ transcription within NK cells are unclear, as FcRγ transcriptional regulation is poorly understood. Recently however, Juang and colleagues reported that Elf-1, a member of the Ets family of transcription factors, is a negative transcriptional regulator of FcRγ in human T lymphocytes whereas it is a positive transcriptional regulator for TCRζ in the same cells [27], [28]. Neither the role of Elf-1 in FcRγ expression in NK cells nor the effect of HIV-1 infection on Elf-1 expression is known. Given the reciprocal effects of Elf-1 on FcRγ and TCRζ expresion, and since we did not observe increased TCRζ expression in both CD56^+^/CD94^+^ lymphocytes and NK cells, we consider this an unlikely mechanism for decreased FcRγ expression.

A limitation of this study is the lack of patients with detectable viremia to enable a correlation of FcRγ expression with viral load to be performed. This was due, in part, to the difficulty in isolating sufficient NK cells from HIV patients with high viral loads to allow biochemical analysis of FcRγ protein levels. This is due to the low numbers of NK cells in such individuals [17]. Firstly, although the sample size was small, the decrease in FcRγ expression in NK cells attained statistical significance and was observed in two groups of patients in CD56^+^/CD94^+^ lymphocytes and in highly purified NK cells. This highlights the reproducible nature of the observed decrease of FcRγ expression even in patients with undetectable viral load. The cross-sectional study described herein needs to be extended with a longitudinal study to determine the relationship between cART and restoration of TCRζ expression in T cells and NK cells, and the effect of cART on FcRγ expression in monocytes and NK cells.

The reason why FcRγ expression decreases in NK cells but not monocytes is not clear. Neither cell type is infected by HIV-1 to an appreciable extent, and changes in FcRγ expression reflect a bystander mechanism. A possible explanation is that NK cells respond more than monocytes to a soluble factor, such as a cytokine, responsible for these changes. We have demonstrated that HIV-1-infection of monocyte-derived macrophages in vitro leads to defective FcγR-mediated phagocytosis through a bystander mechanism which was associated with FcRγ protein depletion [16]. Therefore it is of interest to investigate soluble factors that mediate FcRγ suppression in NK cells, such as TGF-β1 [29], [30]. Alternatively, if changes in FcRγ expression are due to persistent immune activation it would be relevant to correlate loss of FcRγ with plasma endotoxin levels and markers of immune activation such as HLA-DR and CD38 expression.

Our finding that FcRγ is reduced in chronically-infected HIV-infected persons receiving cART suggests that ITAM signalling and function of the NK cell population is defective in the setting of treatment with cART. It is known that elevated plasma endotoxin levels and immune activation are not fully normalised by cART, therefore it is possible that these are linked to aberrant NK cell ITAM signalling and function.
